# Editorial: Microbial Life Under Stress: Biochemical, Genomic, Transcriptomic, Proteomic, Bioinformatics, Evolutionary Aspects, and Biotechnological Applications of Poly-Extremophilic Bacteria, Volume II

**DOI:** 10.3389/fmicb.2022.910962

**Published:** 2022-05-26

**Authors:** Davide Zannoni, Claudia Saavedra, Gloria Levicán, Martina Cappelletti

**Affiliations:** ^1^Department of Pharmacy and Biotechnology, University of Bologna, Bologna, Italy; ^2^Facultad de Ciencias de la Vida, Universitad Andres Bello, Santiago, Chile; ^3^Departamento de Biología, Facultad de Química y Biología, Universidad de Santiago de Chile (USACH), Santaigo, Chile

**Keywords:** environmental stress, molecular mechanisms, extremophiles, adaptive evolution, resistance

Habitats defined as “extremes” exist across the entire planet. They can be widely different in their physico-chemical features as they include a diverse array of harsh parameters thought to preclude the existence of living organisms, such as temperature, pH, salinity, radiation, pressure, low water activity, low nutrients, and even the presence of toxic agents such as metals and/or metalloids. Organisms capable of surviving or thriving in those habitats are named “extremophiles” and the vast majority of them are prokaryotes, which is not surprising as they show a remarkable reservoir of genomes allowing them to grow in a great variety of hostile niches. Interestingly, several harsh conditions may occur simultaneously and the microorganisms able to withstand them are called “poly-extremophiles”. Although many bacterial species from all kinds of extreme environments have been isolated and described in the last decades, very little is known about the molecular strategies and physiology that allow them to grow in such critical conditions.

The aim of this Research Topic on Microbial Life Under Stress (Volume II) is to address this issue by applying multidisciplinary approaches for integrating data from biochemical, genomic, transcriptomic, proteomic, bioinformatics, and evolutionary studies of bacteria from extreme and poly-extreme environments. This Research Topic consists of 14 original articles by numerous authors actively engaged in the study of microbiology, biochemistry, and omic-research of extremophiles. The present Editorial can be divided into four sections which include groups of articles on different genera and species of acidophiles, studies on microorganisms from arid/desiccated environments but also from habitats at low and high temperatures, and finally, a set of papers on extremophiles capable of coping with extreme levels of radiation, pressure, and toxic metals.

Sulfate-reducing bacteria (SRB) are polyextremophiles as they grow at low-pH, high-metal concentration, and high-salinity conditions. These microorganisms are known to catalyze the precipitation of metals in the form of their sulfides and to neutralize the acidity of low pH-drainage environments through proton consumption. In their research article, Sanchez-Andrea et al. reports and describes the isolation of a novel genus of moderately acidophilic SRB, *Acididesulfobacillus acetoxydans* gen. nov. sp. nov. strain INE, able to grow at pH 3.8. Bioreactor studies show that strain INE is capable of completely oxidizing organic acids to CO_2_, a rare property among acidophilic SRB, while comparative proteogenomics and membrane lipid analysis indicate the presence of saturated ether-bound lipids as a protection mechanism against acid stress.

In another paper by Gonzalez-Rosales at al., integrative genomics was applied to investigate the evolution of extreme acidophiles belonging to the Acidithiobacillia class. Phylogenetic reconstruction revealed the genes and mechanisms, defined as first and second lines of defense, which are involved in the exclusion and/or extrusion of protons along with the biosynthesis of hopanoids for membrane stabilization, that are crucial in explaining the acidophilic lifestyle of these microorganisms.

*Acidihalobacter* is a genus of acidophilic, Gram-negative bacteria, known for its ability to oxidize pyrite minerals in the presence of high amounts of chloride ions, a rare property among iron-sulfur oxidizing acidophiles. A phylogenetic analysis of four genomes of this genus *(A. prosperus* DSM 5130Y, *A. yilgarnensis* DSM 105917T, *A. aeolians* DSM 14174T, and *A. ferrooxidans* DSM 14175T) performed by Boase et al. shows that they all root to the Chromatiales class consisting of mostly halophilic microorganisms. The study provides evidence that a series of acid tolerance mechanisms found in Acidihalobacter genomes were acquired by horizontal gene transfer (HGT) from other acidophilic lineages, allowing the genus members to cope with acid stress. These mechanisms include multiple potassium transporters, chloride/proton antiporters, glutamate- and arginine-decarboxylase systems, and both squalene and hopanoid synthesis.

Large-scale outdoor cultivation of autotrophic microalgae is strongly favored by extreme environmental conditions, e.g., very low pH, in order to prevent contamination and predation. In search for new extremophilic microalgae suitable for large-scale production, Abiusi et al. examined six microalgal strains of the genera *Stichococcus, Chlamydomonas, Viridiella*, and *Galdieria*, which are able to grow at pH <3. Several strains were cultivated at variable light intensities and acidic pHs and their autotrophic biomasses were compared with one of the most productive microalgae, *Chlorella sorokiniana* SAG 211-8K grown at pH 6.8. Notably, five of the tested strains displayed autotrophic biomass productivities slightly (10%) or moderately (39%) lower than *C. sorokiniana* indicating the potential use of these microalgae for biomass production under acidic conditions.

Microbiological soil crusts are communities of microorganisms on the soil surface of arid and semi-arid ecosystems. They perform important ecological roles including carbon fixation, nitrogen fixation, and soil stabilization. The paper by Jiang et al. describes the properties of *Modestobacter deserti* sp. nov., a novel phosphate-solubilizing actinobacterium isolated from soil crust samples of the Tengger Desert, China. Whole-genome analysis revealed the presence of a set of genes coding for alkaline phosphatases, phosphate transport, trehalose-phosphate synthase, and trehalose 6-phosphatase along with other genes helping this microorganism to cope with harsh conditions prevalent in deserts. In this respect, the Salar of Atacama in the Chilean Central Andes harbors unique microbial ecosystems due to the high altitude, low oxygen pressure, high solar radiation, and high salinity. The paper by Vignale et al. expands our knowledge of these extreme habitats, through the characterization of 20 previously unexplored Andean microbial ecosystems (both prokaryotic and eukaryotic taxa) in eight different lakes and wetlands. Conversely, Fuentes et al. examined the variation in microbial communities down to a depth of 3.4 m in the hyperarid Yungay region of the Atacama Desert. They found that the moisture content changed from 2 to 11% with depth, with different distributions of bacterial phyla in three zones, namely: A. *Deinococcota, Halobacterota*, and *Latescibacterota*; B. *Crenarchaeota* and *Fusobacteriota*; and C. *Fervibacteria* and *Campilobacterota* (at 0–60 cm, 60–220 cm, and 220–340 cm, respectively). It was concluded that the moisture content, total carbon, pH, and electric conductivity were the best predictors of microbial richness and diversity while total sulfur and phosphorus contents were predictive of community compositions.

In recent years, shallow water hydrothermal vents have been explored for investigating the effects of ocean acidification on microbial communities as vent emissions can release high amounts of CO_2_, causing local pH reduction. However, this phenomenon is often blurred by the emission of other gas species and metals, making this environment highly dynamic. Sciutteri et al. examined the composition and functional profiles of microbial biofilms in Levante Bay (Vulcano Island, Italy, Mediterranean Sea), a well-studied shallow-water hydrothermal vent. Analysis of 16S rRNA transcripts revealed that the composition of the microbial populations varies at very small spatial scales, reflecting the strong influence of H_2_S gas emission as a selection factor for sulfide-dependent and chemolithoautothrophic bacteria. Another example of an extreme marine environment is represented by deep ocean waters and Arctic/Antarctic areas where the temperature is below 0°C. These habitats are colonized by psychrophiles, which are microorganisms thriving at temperatures around the freezing point of water. *Colwelia psychrerythraea* 34H is a Gram-negative psychrophile, isolated from Arctic marine sediments and is considered a model organism for studying adaptive strategies at low temperatures. The paper by Casillo et al. reports that *C. psychrerythraea* generates a series of unusual membrane and extracellular matrix glycopolymers in response to temperature fluctuations. The effects of climate change on environmental microorganisms and in particular on the increase in temperatures in the polar regions were addressed by Garcia-Descalzo et al. who performed a comparative proteomic analysis of psychrophilic (*Shewanella frigidimarina* and *Psychrobacter frigidicola*) vs. mesophilic (*Shewanella oneidensis*) bacterial species. The results show a greater versatility of acclimatization for the genus *Shewanella* with respect to *Psychrobacter*. Furthermore, chaperons play a pivotal role in withstanding temperature changes as they form complexes with other proteins, thus establishing a cooperation with transmembrane proteins, elongation factors, and proteins acting against oxidative damage. The effects of temperature as an abiotic stress factor at the transcriptional level were examined by Guo et al. in *Rhodosporidium kratochvilovae* YM25235, a cold-adapted oleaginous yeast strain that can grow at 15°C. They discover that temperatures around 10–15°C induce substantial changes in the expression of different genes in many fungal genera as part of the process known as the cold-shock response. Comparing the RNA transcriptomic data at 15 and 30°C, ~1,300 differentially expressed genes (DEGs), primarily related to metabolic and cellular processes, cell organelles, and catalytic activities, were identified, providing crucial information on the use of this organism for the production of polyunsaturated fatty acids.

Microbes colonizing the deep seas and oceans represent a large fraction of the biosphere. Therefore, shedding light on their metabolic processes is helpful for understanding global energy cycling. The paper by Zhang et al. that *Iocasia fonsfrigidae* NS-1 gen. nov., sp. nov., a novel Gram-negative anaerobe isolated from deep-sea cold seeps in the South China Sea, metabolizes multiple carbohydrates including xylan, alginate, starch, and lignin. Their anaerobic metabolism generates fermentation products such as hydrogen, lactate, butyrate, and ethanol. The evidence presented predicts that *Iocasia* sp. could be a metabolic partner of other cold-seep microorganisms thriving at the expense of these small macromolecules.

The bacterium *Deinococcus radiodurans* shows remarkable resistance to a range of damage caused by ionizing radiation, desiccation, UV radiation, and oxidizing agents. Although it is known that these multiple resistance phenotypes stem from efficient DNA repair processes, the mechanisms underlying these extraordinary repair capabilities remain poorly understood. Recently, a protein homolog containing a metalloenzyme domain (DRJAMM) with Zn^2+^-dependent deubiquitase activity was reported in *D. radiodurans*. In their paper, Cai et al. show that DRJAMM has efficient *in vitro* catalytic activity in the presence of Mn^2+^, Ca^2+^, Mg^2+^, and Ni^2+^ in addition to Zn^2+^ along with a strong adaptability to a wide range of temperatures. Furthermore, DRJAMM was shown to counteract oxidant processes and it was also required for anaerobic DMSO-respiration.

Bioleaching of copper sulfides is a crucial aspect of iron- and sulfur-oxidizing acidophilic microorganisms in the heap/dump mining industry. The paper by Bobadilla and Poblete-Castro examines in detail the dynamics of biofilm formation using *Leptospirillum* spp. and *Acidithiobacillus thiooxidans* (in a ratio of 3:1) during bioleaching of primary copper sulfide minerals (bornite, Cu_5_FeS_4_ and chalcopyrite, CuFeS_2_) at 30°C. The results show that *A. thiooxidans* cells are able to develop as a biofilm on the surface of bornite while *Leptospirillum* spp. are detected in planktonic form, underlying the role of biofilm formation in the bioleaching process of copper sulfides.

Finally, I would like to mention, on behalf of the co-editors Claudia Saavedra, Gloria Levicán, and Martina Cappelletti, that this Research Topic on Microbial Life Under Stress, is dedicated to the memory of my friend and colleague Claudio Vasquez who died prematurely in July 2020. The remembrance I have of Claudio is that of a generous, authentic, outgoing, and lively man. He was a lover of life and good foods, and, last but not least, a scientist who raised a group of excellent young researchers in his laboratory in Santiago, Chile. My biggest regret is not having had many more opportunities to meet him but for sure Claudio will remain in my thoughts for years to come.

## Author Contributions

DZ wrote the first draft of the manuscript. DZ, CS, GL, and MC contributed to the final version of the manuscript. All authors contributed to the article and approved the submitted version.

## Funding

This work was supported by Fondecyt grant N° 1211386 from the government of Chile (to GL), by Fondecyt 1210633 (to CS) and by RFO-2021 UniBo (to MC).

## Conflict of Interest

The authors declare that the research was conducted in the absence of any commercial or financial relationships that could be construed as a potential conflict of interest.

## Publisher's Note

All claims expressed in this article are solely those of the authors and do not necessarily represent those of their affiliated organizations, or those of the publisher, the editors and the reviewers. Any product that may be evaluated in this article, or claim that may be made by its manufacturer, is not guaranteed or endorsed by the publisher.

